# Feasibility of Somato-Cognitive Coordination Therapy Using Virtual Reality for Patients with Advanced Severe Parkinson’s Disease

**DOI:** 10.3233/JPD-240011

**Published:** 2024-06-04

**Authors:** Masahiko Hara, Yuichiro Murakawa, Tomomi Wagatsuma, Keito Shinmoto, Masatake Tamaki

**Affiliations:** a Department of Neurology and Clinical Rehabilitation, mediVR Rehabilitation Center, Toyonaka, Japan; b Centre for Community-Based Healthcare Research and Education, Shimane University Faculty of Medicine, Izumo, Japan

**Keywords:** Virtual reality, Parkinson’s disease, motor coordination

## Abstract

This feasibility study enrolled 20 patients with advanced severe Parkinson’s disease (PD) to evaluate somato-cognitive coordination therapy (SCCT) using virtual reality. Focusing on the safety and tolerability of SCCT, 17 patients (76±9 years old and 64.7% male) completed the 3-month trial. Key observations included absence of adverse events and tolerability of the participants to SCCT despite initial apprehensions and minor adjustments in medication. Physical functions showed no significant deterioration, suggesting the safety of SCCT. In conclusion, SCCT emerges as feasible and well-tolerated intervention in advanced severe PD, requiring further research to assess its therapeutic efficacy.

## INTRODUCTION

Advanced severe Parkinson’s disease (PD), classified as Hoehn– Yahr severity classification (H&Y) stage IV or V disease, is debilitating [1, 2]. Various treatment options are available for severe PD; however, managing symptoms in the advanced stages remains a significant challenge [3, 4]. Recent technological advancements have led to the exploration of additional rehabilitation approaches, including virtual reality (VR) [5–7]. In this evolving field of VR rehabilitation, we developed a novel intervention approach, named somato-cognitive coordination therapy (SCCT), to facilitate motor coordination by focusing on the involuntary movement of joints other than those intended to be mobilized [8–11].

## METHODS

### Study population and design

We preliminary conducted the single-arm, multicenter, prospective feasibility study enrolling 20 patients with advanced severe PD at three nursing homes. The eligibility criteria were patients for whom both pharmacological treatments and standard rehabilitation activities can only maintain their current physical functions at best in the past 3 months. Exclusions were based on unsuitability for VR therapy including severe mental disorders, as determined by the attending physician. The objective of the study was to evaluate the feasibility of the SCCT approach in this patient population. The primary endpoints were set as the safety and tolerability of SCCT. We have defined “safety” as the absence of adverse events directly related to the SCCT sessions and “tolerability” as the ability of participants to complete the prescribed therapy sessions without discontinuation due to discomfort or other VR-related problems. We also evaluated the Timed Up and Go (TUG) test for gait [12], the Simple Test for Evaluating Hand Function (STEF) for hand function [13], Movement Disorder Society-unified Parkinson’s disease rating scale (MDS-UPDRS) Part 3, and functional independence measure (FIM) as secondary endpoints [1–3]. The purpose of including these secondary measures was not primarily to assess therapeutic efficacy but to ensure that the SCCT did not have detrimental effects on physical functions. The study protocol complied with the principles of the Declaration of Helsinki and was approved by the Central Institutional Ethics Committee of the Japan Society of Clinical Research (UMIN CTR: UMIN000041770). Written informed consent was obtained from all participants.

### Protocol of therapy

**Fig. 1:**
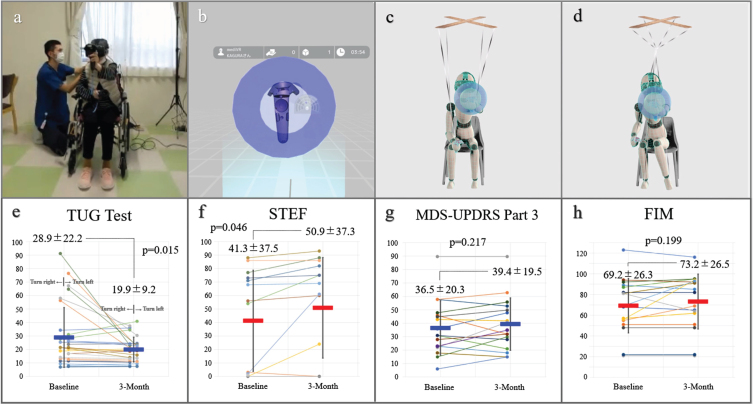
mediVR KAGURA-Guided Somato-Cognitive Coordination Therapy. The patient treatment scene (a) and an image displayed on a device (b). Schematic illustrations of healthy reaching motion (c) and the entangled somato-cognitive action network in a patient (d). After 3 months, there were no significant deteriorations in the Timed Up & Go (TUG) test scores (e), the simple test for evaluating hand function (STEF) score (f), Movement Disorder Society-unified Parkinson’s disease rating scale (MDS-UPDRS) Part 3 (g), and functional independence measure (FIM) (h). Permission was granted by the patient.

We used a commercially available VR device (mediVR KAGURA, mediVR, Inc., Toyonaka, Japan) specialized for SCCT backed by 18 patented technologies [8–11]. In an immersive VR environment deliberately designed without body avatars (Fig. 1a, b), we specifically focused on joint movements outside the intended action, which we termed as articular linkage (AL). This unique VR setting prominently reveals or provokes AL, allowing us to observe the entangled somato-cognitive action network (SCAN) [14]. We conceptualized this entanglement of SCAN, akin to a marionette puppet’s strings becoming tangled, rendering it unable to move one part of its body independently as illustrated in Fig. 1c and 1d. This is a unique perspective that our team has developed [8–11], offering a novel lens through which to view and address the complexities of motor coordination in PD. SCCT was conducted in 20-min sessions, three times a week for 3 months, focusing on reducing AL during tasks. In SCCT sessions, a key feature was having patients alternate reaching tasks between the left and right hands while in a seated position [8–11] (Supplementary Video 1). Patients performed reaching tasks toward static or falling targets in VR space. Therapist adjustments personalized the tasks to each patient’s ability, with successful interactions confirmed by multisensory biofeedback: visual (text), auditory (sound), and tactile (vibration). Sessions focused exclusively on these alternating reaching tasks. This approach aimed to simulate the shifting of body weight experienced during walking, thus potentially enhancing gait abilities in our theory. Additionally, by ensuring trunk stability, we hypothesized an improvement in upper limb functionality without the need for task-oriented training. This method reflects our strategic approach to simultaneously address gait and upper limb functions in advanced PD patients [8–11]. During the study period, attending physicians were requested to minimize any adjustments to medications.

## RESULTS

Seventeen of 20 patients completed the 3-month trial successfully. One participant dropped out owing to relocation, and two could not continue the trial owing to health complications, specifically the common cold and bacterial pneumonia. The mean age was 76±9 years, 11 (64.7%) were male, and the average duration from diagnosis to study enrolment was 11±6 years. Thirteen patients (76.5%) had H&Y stage IV, whereas four (23.5%) had stage V disease. Despite initial apprehensions, all participating patients adapted well to the VR tool with no dropouts due to tolerance issues. In addition, no adverse events, such as VR-induced sickness or falls, occurred during the study period. Regarding secondary endpoints, pre- and post-trial evaluations were possible for 15 using the TUG test (Fig. 1e and Supplementary Video 2) and 14 using STEF (Fig. 1f and Supplementary Video 3). For the TUG test, participants who were unable to walk, even with the use of assistive devices such as walkers, were excluded (*n* = 2) from this portion of the assessment. About the STEF, the inability to complete the transfer of any object was considered a test failure (*n* = 3). All measurements were taken under consistent conditions at the same location and time slot during a presumed best-on time, using the same walking aid. Although we could not make any reliable statements about the effectiveness in this small feasibility study without a control group, there were no significant deteriorations in secondary endpoints. As for the medication status, there were minor adjustments in drug dosages but was no significant difference in levodopa equivalent dose from 512±238 mg at baseline and 518±243 mg at 3 months (*p* = 0.543). We also observed no deterioration in the MDS-UPDRS Part 3 (36.5±20.3 vs. 39.4±19.5, *p* = 0.217) (Fig. 1g) and the FIM scores (69.2±26.3 vs. 73.2±26.5, *p* = 0.199) (Fig. 1h).

## DISCUSSION

Key observations from our study included the safety and tolerability of the participants to VR-guided SCCT. Despite initial apprehensions, all patients expressed enjoyment and willingness to continue post-session resulting in study completion in 17 patients. The absence of adverse events, such as VR-induced sickness or falls, further supports the safety of SCCT. This aspect is particularly important given the vulnerability of the study population for whom managing symptoms remains a significant challenge [1–3]. The primary focus on feasibility, rather than efficacy, is crucial given the study’s design and limitations, such as the small sample size and lack of a control group. In addition, secondary endpoints did not show significant deterioration, emphasizing the safety of the SCCT. The statistical improvements observed in the TUG and STEF scores seem to be driven by partial data points (Fig. 1e, f), raising concerns that these may be the result of statistical anomalies or potentially attributable to a placebo effect. We speculated that minor adjustments in medication dosages did not significantly impact the overall treatment regimen.

### Conclusion

SCCT appears to be a feasible and well-tolerated intervention for advanced severe PD, warranting further research with larger sample sizes and control groups to evaluate its therapeutic efficacy.

## Supplementary Material

Supplementary Material

Supplementary Video 1

Supplementary Video 2

Supplementary Video 3

## Data Availability

The data supporting the findings of this study are available from the corresponding author upon reasonable request.
