# Supplementary material for: Feasibility of Somato-Cognitive Coordination Therapy Using Virtual Reality for Patients with Advanced Severe Parkinson’s Disease
Source: J Parkinsons Dis. 2024 Jun 4;14(4):895–8. doi: 10.3233/JPD-240011 (PMC11191467; doi:10.3233/JPD-240011)
Supplement: Supplementary Material [file jpd-14-jpd240011-s001.pdf]

Supplementary Material

Feasibility of Somato-Cognitive Coordination Therapy Using Virtual Reality for Patients with Advanced Severe Parkinson's Disease

Supplementary Video 1. Alternating Reaching Tasks of mediVR KAGURA-Guided Somato-Cognitive Coordination Therapy. This video showcases a key feature of somato-cognitive coordination therapy where patients in a seated position, engage in alternate reaching tasks with their left and right hands. Patients had to touch blue objects with their left hand and red objects with their right hand using controllers. After successfully touching a target object, patients received multisensory feedback: textual information as visual feedback, a high-pitched "beep" as auditory feedback, and vibration of the controller as tactile feedback.
Video file is available online: https://bit.ly/49ZzC11
Permission was granted by the patient.

Supplementary Video 2. Representative Improvements in Timed Up & Go Test Scores in Patients with Severe Parkinson's Disease. This video shows three cases of patients with severe Parkinson's disease (PD). The first (upper) case involves a 70-year-old male patient with PD of Hoehn–Yahr severity classification (H&Y) stage IV, whose TUG score improved from 67.3 s to 30.3 s. The second (middle) case involves a 72-year-old male patient with PD of H&Y stage V, whose TUG score improved from 64.7 s to 22.9 s. The final (lower) case involves an 89-year-old female patient with PD of H&Y stage IV, whose TUG score was reduced from 21.5 s to 15.8 s. This video provides compelling visual evidence of mediVR KAGURA-guided somato-cognitive coordination therapy in patients with severe PD.
The video file is available online: https://bit.ly/3IH2n6w
Permission was granted by the patient.

Supplementary Video 3. Representative Enhancements in the Simple Test for Evaluating Hand Function Scores in Patients with Severe Parkinson's Disease. This video shows three cases of patients with severe Parkinson's disease (PD). The first (upper) case involves a 70-year-old male patient with PD of Hoehn–Yahr severity classification (H&Y) stage IV, whose simple test for evaluating hand function (STEF) score significantly improved from 0 to 24. The second (middle) case involved a 72-year-old male patient with PD of H&Y stage V, whose STEF score improved from 2 to 61. The final (lower) case involves a 67-year-old male patient with PD of H&Y stage IV, whose STEF score improved from 54 to 75. This video serves as an engaging visual representation of mediVR KAGURA-guided somato-cognitive coordination therapy in patients with severe PD.
The video file is available online: https://bit.ly/43k1zOt
Permission was granted by the patient.
